# Rotavirus genotype diversity in Tanzania during Rotavirus vaccine implementation between 2013 and 2018

**DOI:** 10.1038/s41598-023-49350-4

**Published:** 2023-12-08

**Authors:** Fausta Michael, Mariam M. Mirambo, Dafrossa Lyimo, Abdul Salehe, Furaha Kyesi, Delfina R. Msanga, Dina Mahamba, Helmut Nyawale, Elizabeth Kwiyolecha, Bernard Okamo, Paul J. Mwanyika, Victoria Maghina, Elice Bendera, Mohammed Salehe, Adolfine Hokororo, Ernestina Mwipopo, Asha C. Khamis, Honest Nyaki, Richard Magodi, Delphius Mujuni, Eveline T. Konje, Betina Katembo, Ritha Wilillo, Stephen E. Mshana

**Affiliations:** 1 Ministry of Health, Immunization and Vaccine Development Program, Dodoma, Tanzania; 2 Department of Microbiology and Immunology, Weill Bugando School of Medicine, Catholic University of Health and Allied Sciences, Mwanza, Tanzania; 3 Ministry of Health, Immunization and Vaccine Development Program, Zanzibar, Tanzania; 4 Department of Paediatrics and Child Health, Weill Bugando School of Medicine, Catholic University of Health and Allied Sciences, Mwanza, Tanzania; 5 Department of Pediatrics and Child Health, College of Health Sciences, University of Dodoma, P.O. Box 395, Dodoma, Tanzania; 6 Department of Biochemistry and Molecular Biology, Weill Bugando School of Medicine, Catholic University of Health and Allied Sciences, Mwanza, Tanzania; 7 Department of Pediatrics and Child Health, Mbeya Zonal Referral Hospital, P.O. Box 419, Mbeya, Tanzania; 8 Department of Pediatrics and Child Health, Muheza Designated District Hospital, Tanga, Tanzania; 9 Department of Pediatrics and Child Health, Bombo Regional Referral Hospital, Tanga, Tanzania; 10 Department of Pediatrics and Child Health, Mwananyamala Regional Referral Hospital, Dar es Salaam, Tanzania; 11 Department of Pediatrics and Child Health, Temeke Regional Referral Hospital, Dar es Salaam, Tanzania; 12 Department of Epidemiology and Biostatistics, School of Public Health, Catholic University of Health and Allied Sciences, P.O. Box 1464, Mwanza, Tanzania; 13 National Public Health Laboratory, Dar es Salaam, Tanzania; 14 World Health Organization, Country Office, Dar es Salaam, Tanzania

**Keywords:** Vaccines, Virology, Viral genetics, Paediatric research

## Abstract

The study aims to determine Rotavirus genotypes between 2013 and 2018 during implementation of ROTARIX vaccine in Tanzania. The analysis of surveillance data obtained between 2013 and 2018 was done to determine circulating genotypes after introduction of Rotarix vaccine. From 2013 to 2018, a total of 10,557 samples were collected and screened for Rotavirus using an enzyme immunoassay. A significant decrease in Rotavirus positivity (29.3% to 17.8%) from 2013 to 2018 (OR 0.830, 95% CI 0.803–0.857, *P* < 0.001) was observed. A total of 766 randomly selected Rotavirus positive samples were genotyped. Between 2013 and 2018, a total of 18 Rotavirus genotypes were detected with G1P [8] being the most prevalent. The G1P [8] strain was found to decrease from 72.3% in 2015 to 13.5% in 2018 while the G9P [4] strain increased from 1 to 67.7% in the same years. G2P [4] was found to decrease from 59.7% in 2013 to 6.8% in 2018 while G3P [6] decreased from 11.2% in 2014 to 4.1% in 2018. The data has clearly demonstrated that ROTARIX vaccine has provided protection to varieties of the wild-type Rotavirus strains. Continuous surveillance is needed to monitor the circulation of Rotavirus strains during this era of vaccine implementation.

## Introduction

Rotavirus has been the leading cause of childhood diarrhea among children below five years of age globally with this diarrhea characterized by severe dehydration and increased mortality^1^. The 2016 global estimate reported 258,173,300 episodes of diarrhea among children younger than 5 years with 128,500 deaths^2^. Furthermore, diarrhea was reported to be the third leading cause of disability-adjusted life years (DALYs) in 2016 and was responsible for 74·4 million DALYs and 40·1 million of those occurred among children younger than 5 years^3^. About 95% of these deaths have been reported in low- and middle-income countries (LMICs). The acute gastroenteritis (AGE) hospitalization has been found to be 38% among children < 5 years of age. Implementation of Rotavirus vaccination programmes has reduced AGE hospitalizations by a median of 67%^4^.

Furthermore, it was noted that without Rotavirus vaccination programme, 83% of children developed at least one episode of Rotavirus associated diarrhea in LMICs. Out of these children with diarrhea, 17.9% were found to develop severe disease requiring hospitalization and intravenous fluids with estimated one death out of 293 cases as a result of Rotavirus associated complications^5^. Rotavirus vaccination programmes has significantly reduced Rotavirus associated complications, however, in the sub-Saharan Africa region about 20–49.9 deaths in every 100,000 children below five years of age are still associated with Rotavirus infection^2^.

Despite other routine preventive measures of diarrhea, vaccination remains the main public health approach to reduce the burden of Rotavirus gastroenteritis (RGE) across the globe. The world health organization (WHO) has approved different types of Rotavirus vaccines which have been introduced in the national immunization programmes in more than 100 countries to date, the approved vaccines includes ROTATEQ, ROTARIX, ROTAVAC and ROTASIIL^6^.

These vaccines have demonstrated variable vaccine efficacy (VE) ranging from 44 to 70% against severe RGE in endemic countries compared to non-endemic countries whereby high VE ranging from 90 to 97% were observed against ROTARIX and ROTATEQ^7^. There is limited data for both ROTAVAC 5D^®^ and ROTASIIL in non-endemic and moderate endemic settings. Generally, all vaccines had a decreased protection against severe Rotavirus diarrhoea diseases with comparable VE (48–57%) against severe RGE in high endemic countries i.e. Africa and Asia^8^.

The monovalent Rotavirus vaccine (ROTARIX G1P [8]) was introduced in Tanzania in December 2012 and its implementation into the national Immunization Program started on January 2013^9^. Since its introduction, there has been a notable reduction in Rotavirus associated diarrhea with less severe form of diarrhea as compared to the pre-vaccination era^10,11^. Rotavirus positivity rate has significantly decreased after the introduction of monovalent Rotavirus vaccine (ROTARIX G1P [8]) from 41 to 14% in Tanga and 58% to 18% in Mwanza^12^.

Rotavirus is one of the genus among 15 genera of the family Reoviridae^13^. Rotavirus has been classified into 7 distinct groups (A to G) and 4 specific subgroups within the group A. Group A Rotaviruses are associated with > 90% of human infections and can be further differentiated using a dual classification system that assigns G and P genotypes based on nucleotide sequence similarities of VP7 and VP4 encoding genome segments, respectively^14^.

Currently, there are 220 genotypes with 28 G genotypes and 39 P genotypes^15^. G1, G2, G3, G4, and G9 account for 90% of genotypes globally and among P types P [4], P [6], and P [8] are most prevalent^16^. Previous reports in Tanzania documented circulating genotypes to be G1P [8], G2P [4], G1P [6], G1P [4], G3P [8], G3P [6], G8P [4], G8P [6], G8P [8], G4P [4], G4P [6], G9P [8] and G12P [6], along with untypable and mixed strains^17^.

Changes in circulating Rotavirus strains have been reported post vaccine introduction in some countries^18–22^. Before introduction of Rotavirus vaccine in Tanzania previous studies in Dar es salaam and Mwanza reported the commonest circulating genotypes to be G1P [8], G1P [6] and G1P [4]^23,24^.

In Tanzania coverage of two doses ROTARIX^®^ for the year 2016, 2017, 2018, 2019, 2020 and 2021 has been 98%, 96%, 97%, 98%, 98% and 103% respectively. With this good coverage there is no detailed report regarding the impact of vaccination on the circulating genotypes. This report documents circulating Rotavirus genotypes in sentinel sites of Tanzania, the information that might be useful for further interventions to reduce Rotavirus gastroenteritis across the country and highlight the impact of vaccination on genotypes circulation.

## Materials and methods

### Study area, design, duration and population

This was a descriptive survey conducted using routine Rotavirus Gastroenteritis Surveillance 8 sentinel sites representing the United Republic of Tanzania namely; Mwananyamala Referral Hospital, Temeke Referral Hospital, Bombo Regional Referral Hospital (Eastern zone), Dodoma Regional Referral Hospital (Central zone), Mawenzi Regional Referral Hospital (Northern zone), Mbeya Zonal Referral Hospital (Southern highlands), Bugando Medical Centre (Lake zone), and Mnazi Mmoja Hospital (Zanzibar) between 2013 and 2018. Surveillance was conducted in accordance with the WHO RGE case definitions and case classifications^25^.

A total of 10,557 samples were collected from children with diarrhea as per WHO protocol. In this surveillance diarrhea was defined according to the WHO guidelines as passage of three or more loose, liquid or watery stools within a 24-h period^26^. Information including site, year of sample collection and other relevant clinical information in line with WHO RGE case definition were recorded followed by clinical examination to establish nutritional and hydration status. All children were admitted and managed as per respective standard hospital guidelines. Stool samples were collected and transported to the laboratory for analysis.

### The sample size, duration and population

This was a descriptive survey conducted using routine Rotavirus case-based surveillance system involving 8 sentinel sites of the United Republic of Tanzania between 2013 and 2018. A total of 10,557 samples from children who met RGE case definition were collected, 2473(23.4%) were positive and out of the positive samples, 766(31%) were randomly selected for genotyping.

### Laboratory procedures and descriptive analysis of the samples

Samples from 6 sentinel sites namely Dodoma Regional Referral Hospital (Dodoma), Mawenzi Regional Referral Hospital (Kilimanjaro), Mbeya Zonal Referral Hospital (Mbeya), Bugando Medical Centre (Mwanza), Bombo Regional Referral Hospital (Tanga) and Mnazi Mmoja Hospital (Unguja) were collected and analyzed in laboratories of the respective hospitals while samples from the remaining sentinel sites (Mwananyamala and Temeke Hospital in Dar es Salaam) were collected and shipped to the National Public Health Laboratory (NPHL) for analysis. Samples were analyzed using enzyme-linked immunosorbent assay as per manufacturer’s instructions (The ProSpecT Rotavirus Mi-croplate kit, Oxoid Ltd., UK). A total of 766 randomly selected positive samples by enzyme immunoassay were selected for genotyping. Samples were stored at -80^0^C before transportation to MRC/UL Diarrhea pathogens Research Unit, University of Limpopo, South Africa for genotyping as previously described^14^.

Briefly, RNA was extracted using Trizol (Life Technologies), as previously described^14^. The extracted RNA was resuspended in 15 mL of RNase-free water. The VP7 gene was reverse transcribed and amplified using plus-sense primer sBeg9 (nucleotides 1–21, 5’-GGCTTTAAAAGAGAGAATTTC-3’) and minus-sense primer End9 (nu-cleotides 1062–1036, 5 -GGTCACATCATACAATTCTAATCTAAG-3’), followed by G genotyping using a cocktail of primers specific to the 7 human Rotavirus genotypes (G1–G4, G8, G9 and G12)^14,27^. Additional primers described else-where^27,28^ were used to confirm G genotypes. The VP4 gene was amplified by RT-PCR using gene specific primers, and P-genotypes determined using primers specific for P [4], P [6], P [8], P [9], and P [10] as documented in the WHO manual of Rotavirus detection and characterization methods^14^.

### Data analysis

Data were obtained from the Immunization and Vaccine Development (IVD) program RGE surveillance system. Descriptive data analysis was done using STATA software version 13 (College Station, Texas, USA). Age was summarized with median (months) with interquartile range (IQR) while categorical variables (sex, site etc.) were summarized by proportions. Kruskal–Wallis equality of population rank test was done to compare variability of median age by sites while logistic regression analysis was done to compare the association between age (continuous) and Sex (reference male) with RGE. A *p* value of less than 0.05 was considered statistically significant.

### Reporting guidelines

The Strengthening the Reporting of Observational Studies in Epidemiology (STROBE) guidelines for cross-sectional studies was adhered to ensure the quality of this study. The authors carefully reviewed the checklist and incorporated all relevant items into the introduction, methodology, results section and discussion.

### Patient and public involvement

Patients and the public were not involved in this research’s design, conduct, reporting or dissemination plans.

### Ethical considerations

This surveillance was undertaken as part of the routine RGE surveillance of the Tanzania Ministry of Health. All laboratory procedures were performed in the accordance with relevant guidelines and regulations of the Tanzania National Public Health Laboratory and the Diarrhoeal Pathogens Research Unit (DPRU) University of Limpopo (Medunsa Campus).

### Informed consent

The Informed consent to provide samples and clinical data was obtained by clinicians during routine patient care using clinical investigation forms in the accordance with standard clinical practice. The electronic database was decoded and contained only the unique identifiers and aggregated data without the personal identifiers. Permission to publish the surveillance data was sought from the joint CUHAS and BMC Research Ethics and Review Committee (CREC).

## Results

### Sociodemographic characteristics and Rotavirus enzyme immunoassay positivity

**Figure 1:**
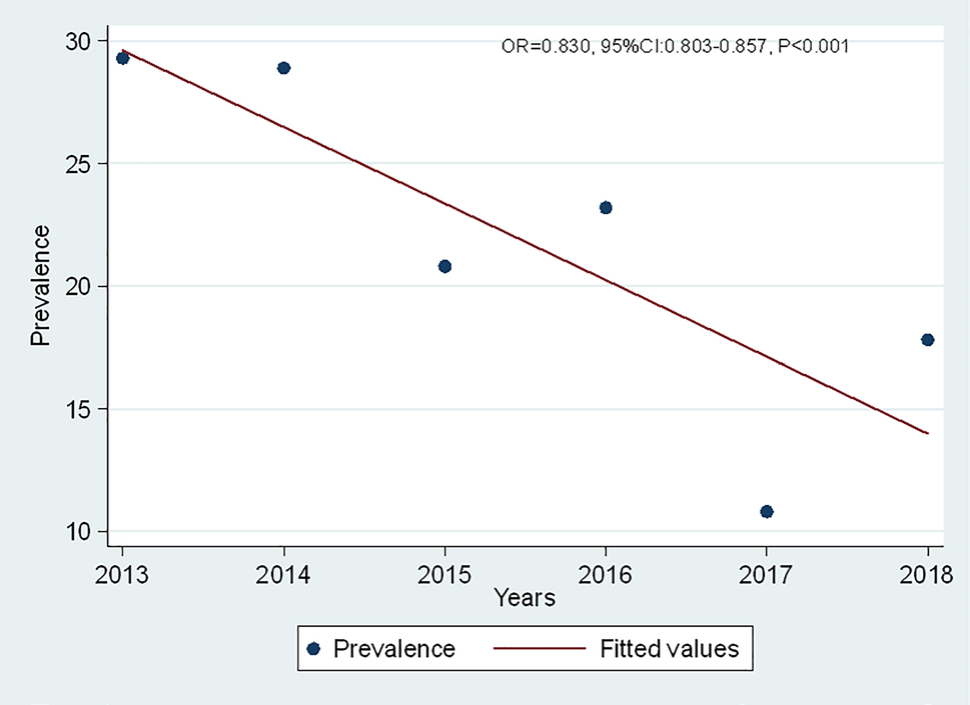
Trend of Positivity by year i.e. 2013: 29.3%, 2014: 28.9%, 2015: 20.9%, 2016: 23.2%, 2017: 10.8% and 2018: 17.8%.

From 2013 to 2018 a total of 10,557 samples from children with gastroenteritis were collected. More than half (6067; 57.5%) of enrolled children were male. The median age of enrolled children was 10 months, interquartile range [IQR]: 7–14 months, with significant variation among sites (Kruskal–Wallis, *P* < 0.001). Out of the collected samples 2473 (23.4%, 95% CI 22.6–24.2) tested positive for the Rotavirus with significant variation among sites (Table 1, Pearson chi^2^ = 135.8, *P* < 0.001). Further analysis showed significant decrease in positivity from 2013 to 2018 (OR 0.830, 95% CI 0.803–0.857, *P* < 0.001) (Fig. 1).

**Table 1:** Samples tested, Rotavirus positivity and median age by site for the period 2013–2018.

Site name	Total sample (n)	Median age (IQR)	Positivity (%)
Bombo Regional Hospital (Tanga)	898	10 (7–14)	208 (23.2)
Bugando Medical Centre (Mwanza)	1695	10 (7–14)	317 (18.7)
Dar es salaam	2131	10 (7–14)	401 (18.8)
Dodoma Regional Hospital (Dodoma)	927	10 (7–15)	302 (32.2)
Mawenzi Referral Hospital (Kilimanjaro)	622	11 (8–16)	136 (21.9)
Mbeya Regional Hospital (Mbeya)	1705	9 (7–14)	517 (30.3)
Mnazi mmoja hospital (Unguja)	2569	10 (7–10)	592 (23.0)
Overall	10,557	10 (7–14)	2473 (23.4)

Overall, using age as continuous variable no significant difference was observed between Rotavirus seropositivity and age (OR 1.003, 95%CI 0.998–1.009, *P* = 0.179). Using male as reference, female children were more likely to have RGE (OR 1.097, 95%CI 1.001–1.202, *P* = 0.047). Except in years 2014 (OR 1.021, 95%CI 1.008–1.034, *P* = 0.001) and 2018 (OR 1.015, 95%CI 1.002–1.028, *P* = 0.023) where there was association between age and RGE no significant association for either age or sex and RGE was observed in all other years (Supplementary file Table 1).

### Genotype distribution

**Figure 2:**
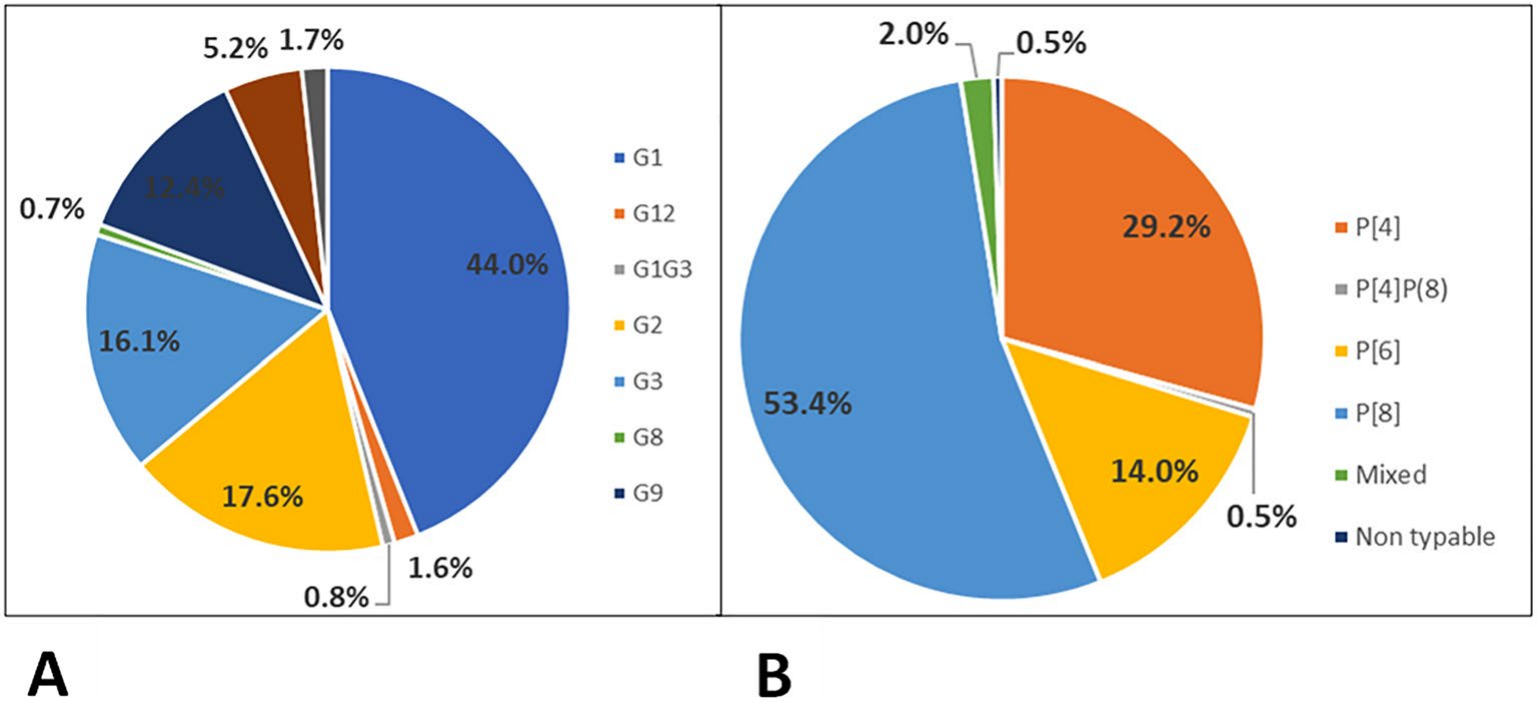
(**A**) The G types observed from 2013 to 2018 in Tanzania sentinel sites (**B**) The P types observed from 2013 to 2018 in Tanzania sentinel sites.

**Figure 3:**
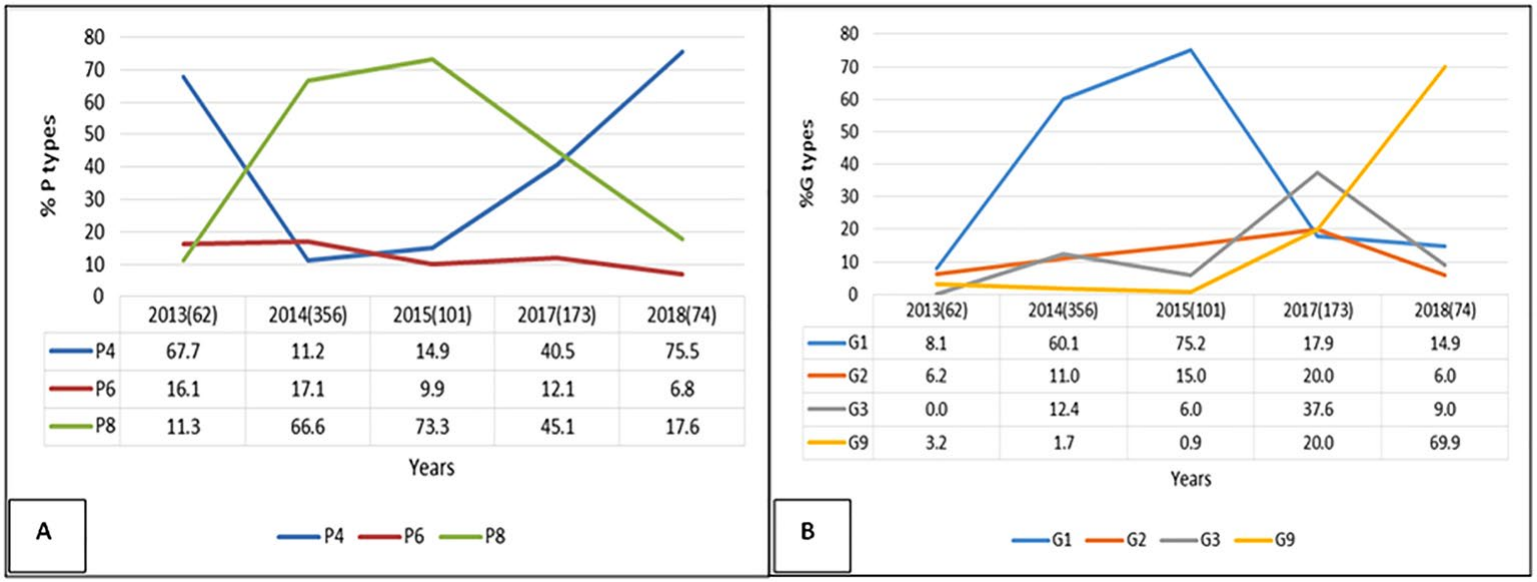
(**A**) and (**B**) showing P [4], P [6] and P [8], and G1 G2, G3 and G9 genotypes trends respectively by years from 2013 to 2018.

Out of 2373 positive samples 766 were genotyped. Overall, in the current study the predominant P types were P [8] (53.4%) followed by P [4] (29.2%) and P [6] (14.0%) (Fig. 2A) while the commonest G types were G1 (44.0%) followed by G2 (17.6%) and G3 (16.1%) (Fig. 2B). The predominance of G1 type was found to decrease from 75.2% in 2015 to 14.9% in 2018 with similar trend in P [8] genotypes which decreased from 73.3% to 17.6% in the same years respectively. G2 and P [6] genotypes showed only minor fluctuations between years (Fig. 3).

Overall, there were 18 genotypes detected from 2013 to 2018. The most frequently detected genotypes were G1P [8] (41.6%), G2P [4] (16.8%), G9P [4] (11.4%) and G3P [6] (8.2%) (Table 2). The prevalence of G1P [8] increased from 8.1% in 2013 to 56.7% in 2014 and thereafter, decreased from 72.3% in 2015 to 13.5% in 2018. Regarding G9P [4], the prevalence decreased from 3.2% in 2013 to 0.8% in 2014 and increased from 1% in 2015 to 67.7% in 2018 (Table 2).

**Table 2:** Rotavirus genotypes by years observed in Tanzania sentinel sites between 2013 and 2018 (n = 766).

SN	Genotype	Number of samples (n %)					Total
		2013	2014	2015	2017	2018	
1	G12P [6]	10 (16.1)	0 (0.0)	0 (0.0)	0 (0.0)	0 (0.0)	10 (1.3)
2	G12P [8]	0 (0.0)	2 (0.6)	0 (0.0)	0 (0.0)	0 (0.0)	2 (0.3)
3	G1G3P [4]	0 (0.0)	0 (0.0)	0 (0.0)	2 (1.2)	0 (0.0)	22 (0.3)
4	G1G3P [6]	0 (0.0)	0 (0.0)	0 (0.0)	4 (2.3)	1 (1.4)	5 (0.7)
5	G1G3P [8]	0 (0.0)	0 (0.0)	0 (0.0)	1 (0.6)	0 (0.0)	1 (0.1)
6	G1P [6]	0 (0.0)	2 (0.6)	1 (1.0)	1 (0.6)	0 (0.0)	4 (0.5)
7	G1P [8]	5 (8.1)	202 (56.7)	73 (72.3)	29 (16.8)	10 (13.5)	319 (41.6)
8	G2P [4]	37 (59.7)	40 (11.2)	16 (15.8)	31 (17.9)	5 (6.8)	129 (16.8)
9	G2P [4] P [8]	0 (0.0)	0 (0.0)	0 (0.0)	4 (2.3)	0 (0.0)	4 (0.5)
10	G2P [6]	0 (0.0)	0 (0.0)	0 (0.0)	1 (0.6)	0 (0.0)	1 (0.1)
11	G3P [4]	0 (0.0)	0 (0.0)	1 (1.0)	6 (3.4)	1 (1.4)	8 (1.0)
12	G3P [6]	0 (0.0)	40 (11.2)	6 (6.0)	14 (8.1)	3 (4.1)	63 (8.2)
13	G3P [8]	0 (0.0)	3 (0.8)	0 (0.0)	45 (26.0)	3 (4.1)	51 (6.7)
14	G8P [4]	0 (0.0)	1 (0.3)	0 (0.0)	0 (0.0)	0 (0.0)	1 (0.1)
15	G8P [6]	0 (0.0)	3 (0.8)	0 (0.0)	0 (0.0)	0 (0.0)	3 (0.4)
16	G9P [4]	2 (3.2)	3 (0.8)	1 (1.0)	31 (17.9)	50 (67.6)	87 (11.4)
17	G9P [6]	0 (0.0)	0 (0.0)	0 (3.0)	1 (0.6)	1 (1.4)	2 (0.3)
18	G9P [8]	0 (0.0)	3 (0.8)	0 (0.0)	3 (1.7)	0 (0.0)	6 (0.8)
19	Missing**	8 (12.9)	57 (15.7)	3 (3.0)	0 (0.0)	0 (0.0)	68 (8.8)
Total genotyped		62	356	101	173	74	766
Total +ve by EIA		419	850	594	193	94	2150*

*323 of the year 2016 were not submitted for genotyping.

**Either G or P types are missing or has mixed Untypable genotypes.

Overall, minor variations of genotype prevalence were observed among sentinel sites. There was more detection of genotype G1P [8] in Dar es Salaam, Dodoma, Mbeya, Unguja and Tanga while genotype G2P [4] was mostly detected in Unguja, Mbeya and Kilimanjaro. The least frequently detected genotypes in all regions were G12P [6], G8P [4], G8P [6] and G9P [6]. Overall, G9P [4] was detected more in Dodoma while G3P [6] was detected more in Unguja (Supplementary File Fig. 2).

## Discussion

The Tanzania government has been implementing two doses of ROTARIX^®^ vaccine since January 2013 with the vaccination coverage the second dose between 85 and 101% during the study period (Supplementary File Fig. 3). The high Rotavirus vaccine coverage has been associated with significant decrease in Rotavirus associated gastroenteritis and associated Rotavirus hospitalizations and death as previously documented in LMICs and high income countries (HICs)^22^. Specifically, the decrease in Rotavirus gastroenteritis and associated complications have been reported in the United Republic of Tanzania as documented in previous studies^10,12,29^.

ROTARIX, a monovalent live attenuated vaccine which contains G1P [8] Rotavirus strain has been found to prevent Rotavirus gastroenteritis caused by G1,G3, G4, and G9 strains in infants and children^30^. The predominant G and P genotypes observed in the current study were G1, G2, G3, G9 and P [8], P [4], P [6] respectively which are similar to the genotypes observed before introduction of ROTARIX vaccine^23,24^. In addition, G1, G4, G8, G9 and G12 were commonly reported in Moshi, Tanzania^31^ after vaccine implementation. Similar unchanged genotype diversity was observed in Malawi before and after introduction of Rotavirus vaccine^32^.

After vaccine implementation, the current genotype data presented in this study between 2013 and 2018 have clearly shown the decrease in G1P [8] genotype confirming the previously reported efficacy of ROTARIX vaccine in preventing G1P [8] RGE^33^. Contrary to what was expected, no changes were observed for G2 and G3 after vaccine implementation. We observed a sharp increase of G9P [4] strains in Dodoma and Mbeya between 2017 and 2018 despite the fact that ROTARIX vaccine has been found to provide cross protection again this strain^34^. Heterotypic cross protection has been observed for the monovalent (G1P [8]) ROTARIX^®^ vaccine in developing countries against completely heterologous strains G2P [4], G8P [6] and G12P [6]^35^. The increase in G9P [4] strain could be due to failure to vaccinate rather than failure of vaccine performance necessitating further studies. In addition, Rotavirus strain ecology could explain this predominance of G9P [4] across most of the sentinel sites as indicative of some epidemiological fitness.

Overall, in the current study G1P [8], G2P [4], G3P [6] were found to be predominant genotypes combinations which is similar to a previous study in Tanzania^36^. However, in 2018 this study observed G9P [4] to be predominant combination in various regions especially Southern highlands and central parts of Tanzania. Further analysis by sentinel sites showed that G1P [8] and G9P [4] were uniformly present in all regions. This implies that there are little regional variations for the commonest genotypes in Tanzania, necessitating longitudinal studies to explore this observation. Furthermore, we observed the high number of untypeable strains accounting for 8.8% of the genotyped strains, highlighting to the possibility of variability of rotavirus strains in the African continent.

In this study the ROTARIX vaccine which is made up of G1P [8] was found to protect number of genotypes circulating in Tanzania except G9P [4] genotype. This could be explained by the expression of serologically or genotypically similar proteins other than those encoded by the various types of G explaining heterotypic protection. Moreover, it has been observed that in the initial exposure to Rotavirus homotypic antibody response is induced, however, with repeated exposure wider heterotypic responses are induced^37–39^. Furthermore, heterotypic protection could be explained by the relative lack of diversity of P types compared to the G types^40^. In addition, cross protection could be due to other immune effector mechanisms other than antibody neutralization^41^. As previously documented in number of studies^42–44^, the current study noted the increase of G9P [4] genotype 5 years after implementation of ROTARIX vaccine. This observation requires further studies to establish if the ROTARIX vaccine has selected this genotype.

### Study limitations

The current study used data collected from 8 sentinel sites which might have limited representation of Tanzania and also 2016 genotyping results were not available. In addition, only 31% of positive samples were genotyped hence there is possibility that some of the important genotypes may have been missed.

## Conclusion

In conclusion, implementation of ROTARIX vaccine with good coverage in Tanzania has resulted in decrease of RGE especially G1P [8] genotype gastroenteritis. The upsurge of G9P [4] genotype during ROTARIX implementation provides strong evidence for the sustained monitoring of Rotavirus strains after vaccine switch. This is timely recommendation due to the fact that from 2022 Tanzania switched from ROTARIX vaccine to ROTAVAC 5D^®^ a monovalent vaccine based on a single live attenuated human G9P [11] strain. Our data suggest the importance of sustaining Rotavirus vaccination to prevent severe Rotavirus gastroenteritis among infants and children.

### Supplementary Information

Supplementary Information 1.

Supplementary Information 2.

Supplementary Information 3.

## Data Availability

All the necessary data are included in the manuscript. Raw data are not publicly available are available upon reasonable request to F.M. fausta.selemani@afya.go.tz.
