# Supplementary material for: Rotavirus genotype diversity in Tanzania during Rotavirus vaccine implementation between 2013 and 2018
Source: Sci Rep. 2023 Dec 8;13:21795. doi: 10.1038/s41598-023-49350-4 (PMC10709589; doi:10.1038/s41598-023-49350-4)
Supplement: Supplementary file 1 — Supplementary Information 1. [file 41598_2023_49350_MOESM1_ESM.pdf]

Supplementary file table 1: Association between Rotavirus positivity, age and sex by year for the period 2013-2018

**Year (n)**	**Age; (OR:, 95% CI:, P value)**	**Sex; OR:, 95% CI:, P value**
2013 (1,831)	1.005, 0.990-1.021, 0.468	1.203, 0.951-1.522, 0.122
2014 (2,935)	1.021, 1.008-1.034, 0.001	1.064, 0.905-1.252, 0.447
2015 (2,845)	1.002, 0.989-1.016, 0.693	1.138, 0.948-1.367, 0.164
2016 (1,397)	1.010, 0.997-1.022, 0.102	0.935, 0.728-1.202, 0.604
2017 (866)	**1**.003, 0.985-1.022, 0.708	1.231, 0.795-1.907, 0.350
2018 (1,883)	1.015, 1.002-1.028, 0.023	0.982, 0.717-1.346, 0.915
Overall (10,557)	1.003, 0.998-1.009, 0.179	1.097, 1.001-1.202, 0.047

**Age was used as continuous variable, Sex: Male was used as reference*
