# Supplementary material for: Rotavirus genotype diversity in Tanzania during Rotavirus vaccine implementation between 2013 and 2018
Source: Sci Rep. 2023 Dec 8;13:21795. doi: 10.1038/s41598-023-49350-4 (PMC10709589; doi:10.1038/s41598-023-49350-4)
Supplement: Supplementary file 2 — Supplementary Information 2. [file 41598_2023_49350_MOESM2_ESM.pptx]

## Slide 1
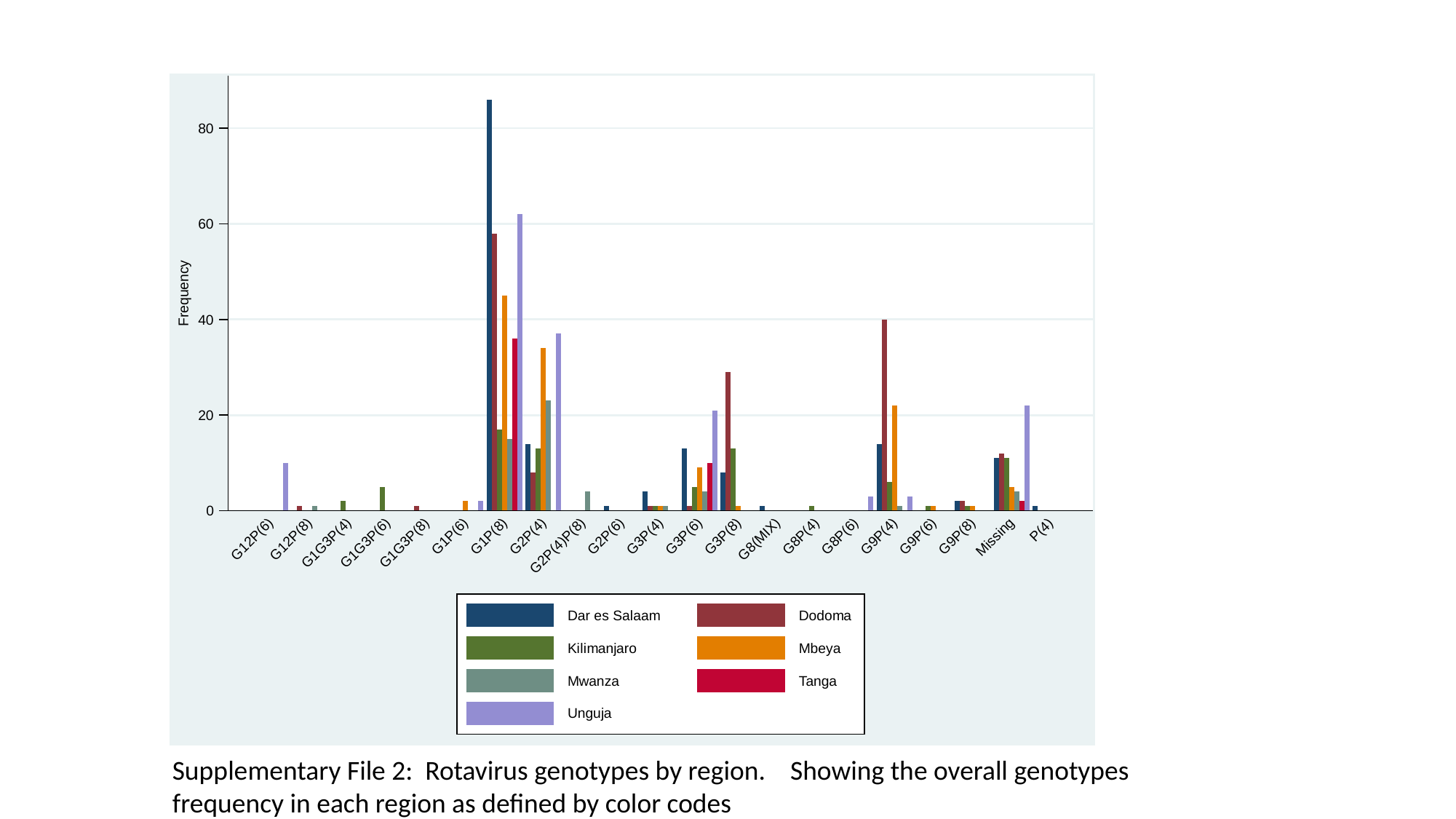

Supplementary File 2: Rotavirus genotypes by region. Showing the overall genotypes frequency in each region as defined by color codes
